# Supplementary material for: The interactome of intact mitochondria by cross-linking mass spectrometry provides evidence for coexisting respiratory supercomplexes
Source: Mol Cell Proteomics. 2017 Dec 8;17(2):216–32. doi: 10.1074/mcp.RA117.000470 (PMC5795388; doi:10.1074/mcp.RA117.000470)
Supplement: Supplemental Data [file supp_RA117.000470_133922_0_supp_23978_fzffwf.zip › spectra_annotation/mito_DR_spectra_annotation/361-1-1-1-4-1.pdf]

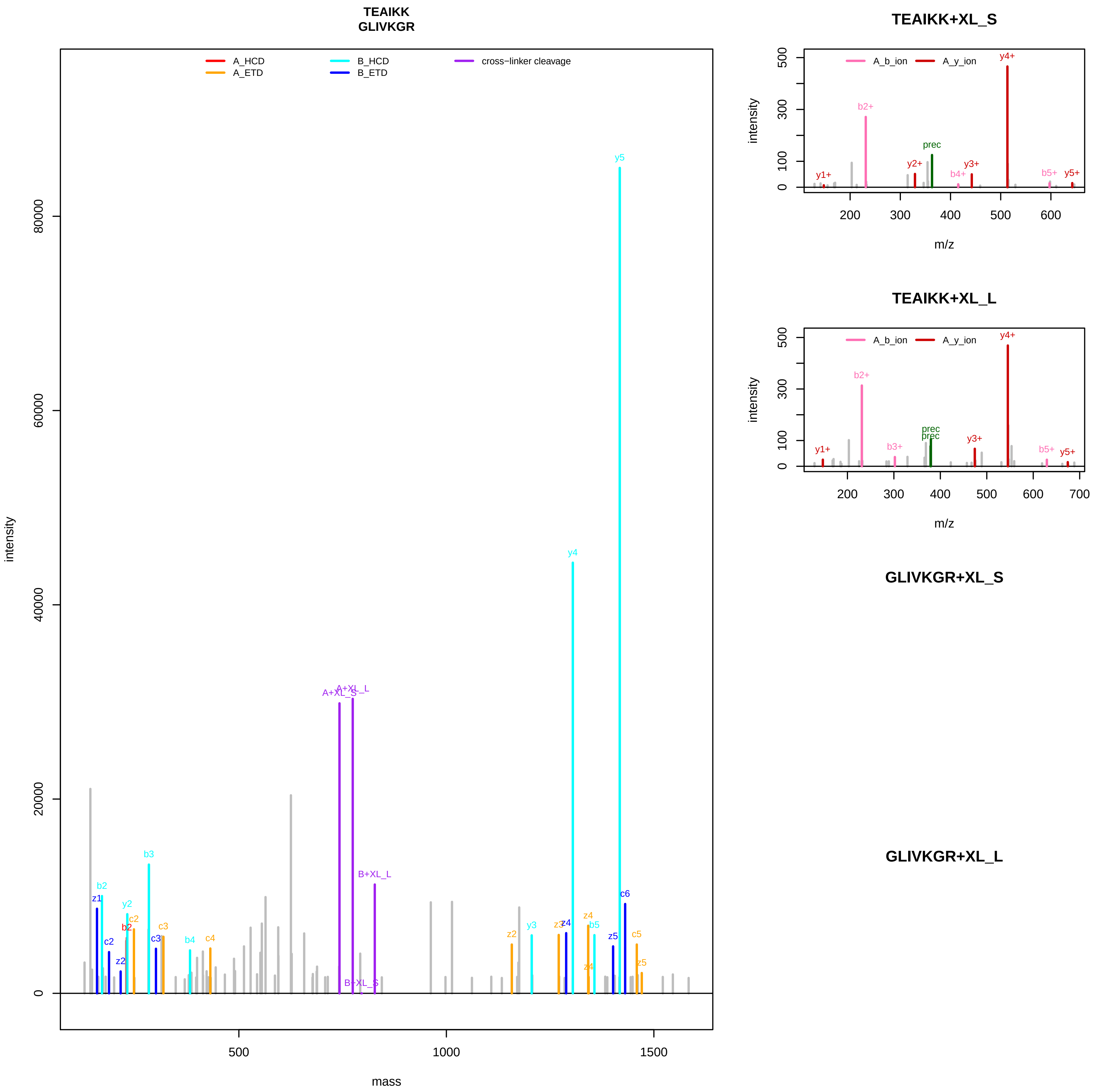
TEAIKK
GLIVKGR
A_HCD
A_ETD
B_HCD
B_ETD
cross−linker cleavage
intensity
mass
0
20000
40000
60000
80000
500
1000
1500
y5
y4
A+XL_S
A+XL_L
B+XL_L
B+XL_S
b2
b3
b4
b5
y2
y3
z1
z2
z3
z4
z5
c2
c3
c4
c5
c6
TEAIKK+XL_S
TEAIKK+XL_L
A_b_ion
A_y_ion
intensity
m/z
prec
y1+
y2+
y3+
y4+
y5+
b2+
b3+
b4+
b5+
GLIVKGR+XL_S
GLIVKGR+XL_L
